# DNA methylation markers associated with type 2 diabetes, fasting glucose and HbA_1c_ levels: a systematic review and replication in a case–control sample of the Lifelines study

**DOI:** 10.1007/s00125-017-4497-7

**Published:** 2017-11-21

**Authors:** Eliza Walaszczyk, Mirjam Luijten, Annemieke M. W. Spijkerman, Marc J. Bonder, Helen L. Lutgers, Harold Snieder, Bruce H. R. Wolffenbuttel, Jana V. van Vliet-Ostaptchouk

**Affiliations:** 1 Department of Epidemiology, University of Groningen, University Medical Center Groningen, Groningen, the Netherlands; 2 Centre for Health Protection, National Institute for Public Health and the Environment (RIVM), Bilthoven, the Netherlands; 3 Centre for Nutrition, Prevention and Health Services, RIVM, Bilthoven, the Netherlands; 4 Department of Genetics, University of Groningen, University Medical Center Groningen, Groningen, the Netherlands; 5 Department of Endocrinology, University of Groningen, University Medical Center Groningen, HPC AA31, P.O. Box 30001, 9700 RB Groningen, the Netherlands

**Keywords:** DNA methylation, Epigenome-wide association studies, Glucose, Glycated haemoglobin, Systematic review, Type 2 diabetes

## Abstract

**Aims/hypothesis:**

Epigenetic mechanisms may play an important role in the aetiology of type 2 diabetes. Recent epigenome-wide association studies (EWASs) identified several DNA methylation markers associated with type 2 diabetes, fasting glucose and HbA_1c_ levels. Here we present a systematic review of these studies and attempt to replicate the CpG sites (CpGs) with the most significant associations from these EWASs in a case–control sample of the Lifelines study.

**Methods:**

We performed a systematic literature search in PubMed and EMBASE for EWASs to test the association between DNA methylation and type 2 diabetes and/or glycaemic traits and reviewed the search results. For replication purposes we selected 100 unique CpGs identified in peripheral blood, pancreas, adipose tissue and liver from 15 EWASs, using study-specific Bonferroni-corrected significance thresholds. Methylation data (Illumina 450K array) in whole blood from 100 type 2 diabetic individuals and 100 control individuals from the Lifelines study were available. Multivariate linear models were used to examine the associations of the specific CpGs with type 2 diabetes and glycaemic traits.

**Results:**

From the 52 CpGs identified in blood and selected for replication, 15 CpGs showed nominally significant associations with type 2 diabetes in the Lifelines sample (*p* < 0.05). The results for five CpGs (in *ABCG1*, *LOXL2*, *TXNIP*, *SLC1A5* and *SREBF1*) remained significant after a stringent multiple-testing correction (changes in methylation from −3% up to 3.6%, *p* < 0.0009). All associations were directionally consistent with the original EWAS results. None of the selected CpGs from the tissue-specific EWASs were replicated in our methylation data from whole blood. We were also unable to replicate any of the CpGs associated with HbA_1c_ levels in the healthy control individuals of our sample, while two CpGs (in *ABCG1* and *CCDC57*) for fasting glucose were replicated at a nominal significance level (*p <* 0.05).

**Conclusions/interpretation:**

A number of differentially methylated CpGs reported to be associated with type 2 diabetes in the EWAS literature were replicated in blood and show promise for clinical use as disease biomarkers. However, more prospective studies are needed to support the robustness of these findings.

**Electronic supplementary material:**

The online version of this article (10.1007/s00125-017-4497-7) contains peer-reviewed but unedited supplementary material, which is available to authorised users.

## Introduction

Type 2 diabetes mellitus is a complex metabolic disease, of which the prevalence worldwide is growing rapidly. According to recent data, globally 415 million people are estimated to have type 2 diabetes [1]. Hallmarks of type 2 diabetes include chronically elevated blood glucose levels due to decreased insulin secretion from pancreatic beta cells and insulin resistance in different tissues [2].

In addition to well-known risk factors for type 2 diabetes such as being overweight, unhealthy lifestyle, metabolic alterations, previous diagnosis of gestational diabetes, or a family history of cardiovascular disease (CVD) or type 2 diabetes [3], genetic susceptibility to the disease is also important, with heritability estimates ranging from 20% to 80% [4, 5]. To date, genome-wide association studies (GWASs) have identified at least 75 loci associated with type 2 diabetes [6]. However, these genetic variants explain only 10–15% of disease heritability, suggesting a major role for environmental and lifestyle factors [6, 7].

To identify the missing component of type 2 diabetes pathogenesis, researchers have started to examine the role of epigenetics in the disease aetiology. Epigenetics refers to DNA alterations that lead to differences in gene expression without changing the DNA sequence. These epigenetic changes can be influenced by the environment and may cause differences in disease susceptibility between individuals [8].

Initially, epigenetic studies used a candidate gene approach to identify DNA methylation changes in known type 2 diabetes susceptibility genes. With the advances in measurement technology, approaches have shifted towards epigenome-wide association studies (EWASs), allowing novel biomarkers for complex diseases to be found. Development of type 2 diabetes requires perturbation of multiple biological mechanisms in different organs, including pancreas, liver, skeletal muscle and adipose tissue [9]. EWASs using those tissues would provide a comprehensive insight into the disease aetiology; however, access to such samples is not possible on a large scale. Therefore, most EWASs have been conducted using whole blood [10].

Here, we present an overview of recent human EWASs investigating DNA methylation changes associated with type 2 diabetes and/or glycaemic traits represented by fasting glucose and HbA_1c_ levels. Moreover, we discuss the EWASs findings and the strengths and limitations of different approaches. To validate methylation loci identified in the reviewed EWASs, we also performed a replication study in blood samples of 100 diabetic and 100 control individuals selected from a Dutch population-based Lifelines study [11]. Next, we investigated whether differential DNA methylation patterns as previously identified in pancreas, liver and adipose tissue were also reflected in blood.

## Methods

### Literature search

**Fig. 1:**
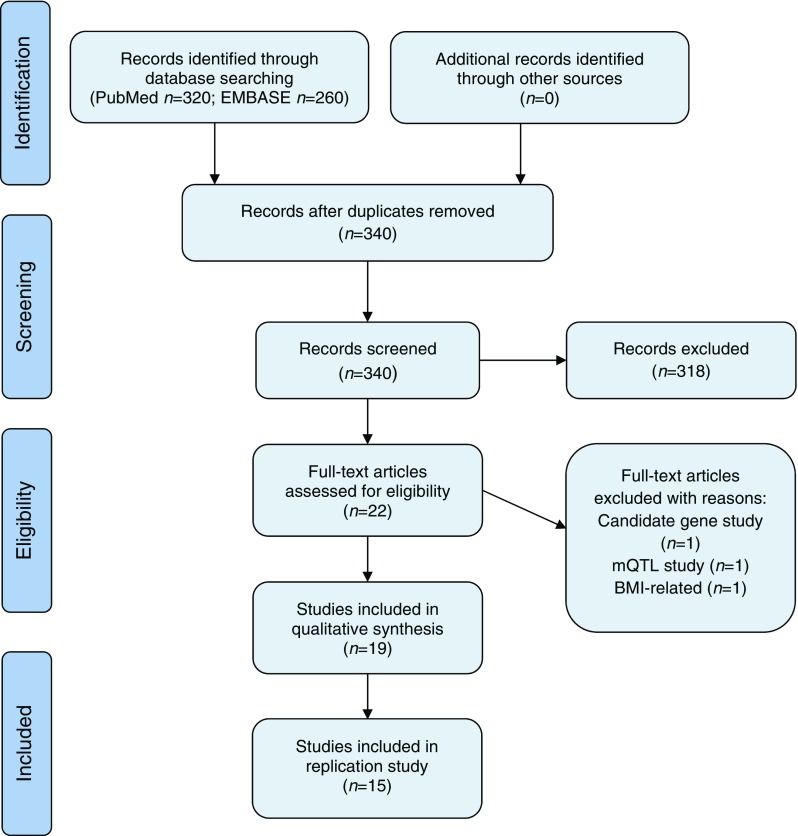
PRISMA 2009 flow chart of the literature search performed up to 26 April 2017

The systematic review was conducted according to the PRISMA and MOOSE guidelines. We searched PubMed and EMBASE for relevant studies investigating DNA methylation associated with type 2 diabetes or fasting glucose and HbA_1c_ levels, up to 26 April 2017. The search strategy, inclusion and exclusion criteria are provided in the electronic supplementary material (ESM Methods). Ultimately, 22 publications were selected for whole-text evaluation. Three studies were excluded (Fig. 1), resulting in a total of 19 studies included in the review.

### Replication analyses: selection of CpG sites

For the replication analyses, four additional studies were excluded: one that only indirectly investigated association with type 2 diabetes [12] and three that used a different platform from the Illumina array [13–15]. Thus, 15 studies were included for replication analysis (Fig. 1). For further CpG sites (CpGs) selection, we applied a study-specific Bonferroni correction for multiple testing for EWASs results (*p* value < 0.05/(the number of CpGs analysed)), even if a different multiple-testing correction was used by the authors of the original manuscript. This was done to avoid false positive results from the studies that used lenient significance thresholds.

### Lifelines case–control sample

Lifelines is a prospective population-based cohort to study health and health-related behaviours of 167,729 individuals living in the North of the Netherlands [16]. Details on clinical examination and biochemical measurements have been described elsewhere [16]. In short, a standardised protocol was used to obtain blood pressure and anthropometric measurements such as height, weight and waist circumference. Blood was collected in the fasting state, between 08:00 and 10:00 h. On the same day, fasting blood glucose and HbA_1c_ were measured.

For this study we used a case–control sample selected from the baseline of the Lifelines study (all unrelated and European ancestry samples, *n* = 13,436) [11]. Four groups were selected based on the following criteria (*n* = 50 for each group):

1. type 2 diabetes patients without CVD complications;
2. type 2 diabetes patients with CVD complications;
3. non-diabetic control participants, with no history of CVD risk factors, and age- and sex-matched to groups 1 and 2;
4. healthy, normal-weight control participants (BMI < 25), additionally obtained from available methylation dataset to increase the power of the study.

In total, we included 100 type 2 diabetic individuals and 100 control individuals. Diagnosis was based on self-reported disease and/or use of blood-glucose lowering medication, or an elevated fasting blood glucose ≥ 7.0 mmol/l at examination. Individuals with CVD complications had a CVD history defined as self-reported myocardial infarction, stroke, angina pectoris or vascular intervention.

### DNA methylation methodology

DNA was isolated from fasting whole blood samples. Next, 500 ng of genomic DNA was bisulphite modified using the EZ DNA Methylation kit (Zymo Research, Irvine, CA, USA) and hybridised to Illumina 450K arrays (San Diego, CA, USA) according to the manufacturer’s protocols. Data were generated by the Genome Analysis Facility of UMCG, the Netherlands (www.rug.nl/research/genetics/genomeanalysisfacility/). Quality control (QC) and normalisation steps are described in detail elsewhere [17] and in ESM Methods. In short, the QC pipeline developed by Touleimat and Tost was used with background correction and probe type normalisation [18]. Then, normalised β values were logit-transformed into M values for downstream analysis, since they have been shown to perform better in studies with smaller sample sizes [19].

### Statistical analysis

All analyses were performed using R-studio software (version 3.3.0; https://www.rstudio.com; https://www.r-project.org) and the limma package. Linear regression model 1 included age, sex, measured blood cell composition (percentage of basophilic granulocytes, eosinophilic granulocytes, neutrophilic granulocytes, lymphocytes and monocytes), plate number and position on the plate as covariates. Additionally, we adjusted for other covariates in models 2–6: (2) model 1 + BMI; (3) model 1 + medication use and newly diagnosed diabetes; (4) model 1 + smoking status and education level; (5) model 1 + presence of cardiovascular complications; (6) model 2 + education level. In addition to the adjustment for measured cell type composition, we estimated cell types based on the Houseman method [20] and compared results. We also performed sensitivity analyses using the model 1 in smaller groups: (1) 50 type 2 diabetes individuals without complications compared only with 50 age- and sex-matched control individuals; and (2) 100 type 2 diabetes individuals with and without complications compared only with 50 age- and sex-matched control individuals. To determine whether the methylation levels at replicated top hits were correlated with type 2 diabetes risk factors, we calculated Pearson correlation coefficients based on methylation β values. We used a strict analysis-specific Bonferroni correction for multiple testing (*p* value < 0.05/(the number of CpGs selected for replication)).

## Results

### Recent discoveries

Our search strategy retrieved 19 EWASs investigating DNA methylation associated with type 2 diabetes or glycaemic traits (Fig. 1), including 16 studies focusing on type 2 diabetes as outcome (Table 1) and four studies focusing on glycaemic traits (Table 2), with one study listed twice [25]. We assessed the quality of included studies using the Newcastle–Ottawa scale for observational studies (details in ESM Methods) [36]. Seventeen out of 20 studies (one listed twice) were assessed to have a low or medium risk of bias and only three studies were evaluated to have high risk of bias (data not shown). In the majority of the reviewed studies, an array-based methodology was employed in the discovery phase: two using the 27K and 13 using the 450K Illumina array. Only one study used whole-genome bisulphite sequencing, which is considered a gold standard in methylation studies [14]. Most of the blood-based studies (ten out of 19) were performed in larger sample sizes (*n* = 6 – Z2000) than studies in pancreas, liver, skeletal muscle and adipose tissue (*n* = 12–100). The EWASs were conducted in different ethnic groups: Europeans, Indian Asians, Mexican Americans, and Ashkenazi Jews [21, 24, 25, 28]. Despite the differences in ethnicity and study design, some CpGs such as those in the *ABCG1*, *TXNIP* and *SREBF1* genes were reported in multiple blood-based studies [21, 23–25, 33, 34]. There was no clear overlap in significant CpGs across tissues, but some studies reported a significant correlation between the level of methylation at specific CpGs in blood and liver [21] or in blood and pancreas [12].

**Table 1:** Characteristics of EWASs associated with type 2 diabetes

Reference^a^	Population for DNA methylation analysis	Female/male	Design	Tissue	Method	Covariates included in analysis	Multiple-testing correction^b^	Top findings	No. of CpGs included in replication study^c^
Chambers et al, 2015 [21]^a^	1074 incident type 2 diabetes patients, 1590 controls (Indian Asians); 1141 Europeans for replication	P 352/722 C 426/1083	Longitudinal (nested case–control)	Blood	450k for discovery; pyrosequencing and 450k for replication	Age, sex, intensity values from 450k control probes, batch, measured and imputed (Houseman method) cell count, 5 PC	Discovery *p* < 5 × 10^−7^ Meta-analysis Bonferroni	5 DMS replicated in independent cohorts: *TXNIP* gene (cg19693031); *SREBF1* gene (cg11024682); *PHOSPHO1* gene (cg02650017); *SOCS3* gene (cg18181703); *ABCG1* gene (cg06500161)	5
Al Muftah et al, 2016 [22]^a^	30 T2D patients, 93 controls from 15 families of Qatari descent; 810 female twins from TwinsUK for replication	72/51	Cross-sectional (case–control)	Blood	450k	Age, sex, smoking status, cell count (Houseman method); BMI as confounder	Bonferroni	1 DMS: *DQX1* gene (cg06721411) replicated in TwinsUK	1
Soriano-Tarraga et al, 2016 [23]^a^	151 T2D patients and 204 controls from IS cohort; BISMAR_2 (59 T2D patients and 108 controls); REGICOR (63 T2D patients and 582 controls) for replication	P 61/90 C 88/116	Cross-sectional (case–control)	Blood	450k	Age, sex, smoking, hyperlipidaemia, BMI, cell count (Houseman method)	Bonferroni	1 DMS: *TXNIP* gene (cg19693031) replicated in 2 independent cohorts	1
Florath et al, 2016 [24]^a^	153 T2D patients and 835 controls; 87 T2D patients and 527 controls for replication	P 56 & 59% male (controlled or poorly controlled T2D) C 49% male	Cross-sectional (case–control)	Blood	450k	Age, sex, BMI, smoking, batch, cell count (Houseman method)	FDR < 5% Bonferroni for replication	39 DMS associated with T2D in discovery cohort, after replication in independent cohort 1 DMS remain significant: *TXNIP* (cg19693031)	1
Kulkarni et al, 2015 [25]^a^	850 pedigreed Mexican Americans (174 T2D patients)	536/314	Cross-sectional (case–control)	Blood	450k	Age, sex, BMI, cell count (Jaffe method)	Bonferroni	Overall 51 DMS associated with T2D status; 19 with fasting glucose level and 24 with HOMA-IR	51
Yuan et al, 2014 [26]^a^	27 monozygotic twin pairs from TwinsUK (17 pairs T2D-discordant, 3 pairs T2D concordant and 7 healthy pairs); 42 unrelated T2D cases and 221 controls for replication	23 pairs/4 pairs	Cross-sectional (twins study)	Blood (white blood cells)	MeDIP-seq 450k for replication	Age, sex, BMI, beadchip, bisulphite conversion efficiency, (family as a random effect)	FDR < 5%	1 DMR: *MALT1* gene (ch18:56336501-56337000), replicated using Illumina 450k array (cg24182998), replicated reached Bonferroni threshold (0.05/20 = 0.0025)	1
Matsha et al, 2016 [13]	3 T2D patients, 3 pre-diabetes, 3 controls (age, sex, BMI and duration of residence matched)	All female	Cross-sectional (case–control)	Blood	MeDIP sequencing	–	q = 10^−2^	450,142 DMRs were statistically significant in all samples, among others associated with cell surface receptor signalling, inflammatory pathways, glucose transport, muscle and pancreas development genes, insulin signalling	0, not an Illumina array
Toperoff et al, 2012 [15]	Ashkenazi Jews: 710 T2D patients and 459 controls were assembled in 4 age-matched pools	Males fraction around 50% in all 4 pools	Cross-sectional with multistep design (case–control)	Blood	Microarray-based assay for methylation levels; sequencing of bisulfite converted DNA pools	Sex and lymphocyte percentage	FDR	6 DMRs were found in LD blocks. After replication and multiple hypothesis testing 13 out of 93 CpGs located in 6 DMRs showed significant case–control difference. Among them the nearest genes were: *THADA, JAZF1, SLC30A8*, *TCF7L2, KCNQ1* and *FTO*. CpG site near *FTO* showed small (3.35%) but significant hypomethylation of cases vs controls	0, not an Illumina array
Bacos et al, 2016 [12]	87 non-diabetic donors for discovery phase and 112 individuals from Danish Family Study	34/53	Cross-sectional association with age	Pancreatic islets and whole blood	450k genome-wide and 4 sites replicated using pyrosequencing in blood	Sex, BMI, HbA_1c_, bisulphite treatment, days in culture and islet purity	FDR < 5%	Ageing was significantly associated with altered DNA methylation at 241 sites in pancreas; almost 60% of sites were found also in other studies in blood; 4 most significant sites (*FHL2*, *ZNF51*8B, *GNPNAT1* and *HLTF*) were selected for follow-up analysis and they showed functional effects on beta cells or association with T2D risk. Higher methylation of those sites was associated with lower risk of T2D development during progression into T2D (mean 10.8 years; Botnia Prospective Study)	0, indirect association with diabetes
Volkov et al, 2017 [14]	6 T2D donors and 8 control donors	P 3/3 C 4/4	Cross-sectional (case–control)	Pancreatic islets	WGBS	–	–	Average methylation level was 75.9%, 25,820 DMRs were identified in T2D pancreatic islets, while 13,696 were hypermethylated and 12,124 were hypomethylated. 692 DMRs had a methylation difference > 10%, the highest in regions annotated to *ARX* and *TFAM* genes	0, not an Illumina array
Dayeh et al, 2014 [27]^a^	15 T2D donors and 34 control donors	P 5/10 C 12/22	Cross-sectional (case–control)	Pancreatic islets	450k	Age, sex, BMI, batch, islet purity, day of culture	FDR < 5%	1649 DMS (853 genes and 561 intragenic) with at least 5% difference in methylation between diabetic and non-diabetic donors	15
Volkmar et al, 2012 [28]^a^	5 T2D donors and 11 non-diabetic donors matched by age, BMI and ethnicity; 12 T2D patients and 12 age- and BMI-matched controls for replication	–	Cross-sectional (case–control)	Pancreatic islets	27k	–	*p* < 0.01 and 15% group-wise difference on methylation level	276 DMS (254 genes) were found, 96% were hypomethylated	0
Nilsson et al, 2014 [29]^a^	14 monozygotic twins discordant for T2D; Cohort 2: 28 T2D patients/28 controls (unrelated)	P 5/9 C 5/9	Cross-sectional (twins study)	Adipose tissue	450k	BMI, glucose	FDR < 15%	In twins 23,470 DMS were found, none passed FDR correction In Cohort 2 15,627 DMS (7046 genes) were found after a FDR correction DNA methylation of 266 sites, corresponding to 103 genes, was significantly associated with expression in the discordant twins at q < 0.15	0
Ribel-Madsen et al, 2012 [30]^a^	12 Danish monozygotic twins discordant for T2D	P 6/6 C 6/6	Cross-sectional (twins study)	Skeletal muscle (11 pairs) Adipose tissue (5 pairs)	27k	–	P_adj_ (Westfall–Young resampling method *p* < 0.001)	1 DMS in skeletal muscle: *IL8* gene and 7 DMS in adipose tissue: *ZNF668*; *HSPA2*; *C8orf31*; *CD320*; *TWIST1*; *MYO5A*	0
Kirchner et al, 2016 [31]^a^	8 obese T2D men, 7 obese non-diabetic controls and 7 non-obese metabolically healthy control individuals	All male	Cross-sectional (case–control)	Liver	450k	–	FDR < 25%	2255 DMS (1388 genes) were found (T2D obese compared with non-obese control individuals)	0
Nilsson et al, 2015 [32]^a^	35 T2D patients and 60 control individuals	P 18/17 C 43/17	Cross-sectional (case–control)	Liver	450k	Age, sex, BMI, NASH diagnosis, degree of steatosis	FDR < 5%	251 DMS (167 genes) were found mostly hypomethylated (94%) in those with T2D. A decrease in folate levels in T2D patients was observed, which could explain decreased methylation in the human liver in diabetes	3

^a^Studies included into replication study to provide CpGs, which passed strict Bonferroni correction threshold

^b^Multiple-testing threshold calculated originally by the authors of particular study

^c^0 in last column means zero CpG sites passed study-specific Bonferroni correction threshold

C, controls; DMS, differentially methylated sites; DMR, differentially methylated region; FDR, false discovery rate; HOMA-IR, homeostatic model assessment; IS, ischemic stroke; LD, linkage disequilibrium; NASH, non-alcoholic steatohepatitis; P, patients; PC, principal component; T2D, type 2 diabetes; 27k, Infinium HumanMethylation27 BeadChip; 450k, Infinium HumanMethylation450 BeadChip

**Table 2:** Characteristics of EWASs associated with glycaemic traits

Reference^a^	Population for DNA methylation analysis	Female/male	Design	Tissue	Method	Covariates included into analysis	Multiple-testing correction^b^	Top findings	No. of CpGs included in replication study^c^
Kriebel et al, 2016 [33] ^a^	1448 non-diabetic individuals (fasting glucose, 2-h glucose, HbA_1c_); 1440 non-diabetic individuals (fasting insulin, HOMA-IR); 617 non-diabetic individuals (2-h insulin)	47.1% male	Cross-sectional	Blood	450k	FDR	Age, sex, cell count (Houseman method), smoking, BMI	Total of 31 CpGs were found to be associated with phenotypic traits: 5 DMS associated with fasting glucose (including *ABCG1, CPT1A*), 1 with 2-h glucose, 8 with fasting insulin, 26 with HOMA-IR and none with HbA_1c_. Using a different model, adjustment for BMI resulted in ~30% reduction in effect size, suggesting BMI had a confounding effect	3 for fasting glucose
Kulkarni et al, 2015 [25] ^a^	850 pedigreed Mexican Americans (174 T2D patients)	536/314	Cross-sectional (case–control)	Blood	450k	Bonferroni	Age, sex, BMI, cell count (Jaffe method)	51 CpGs were significantly associated with T2D, 19 with fasting glucose and 24 with HOMA-IR	19 for fasting glucose
Hidalgo et al, 2014 [34]	544 healthy individuals in discovery stage, 293 in replication stage	286/258	Cross-sectional	Blood	450k	Bonferroni	Age, sex, study site, 4 PC, insulin, glucose	1 DMS associated with fasting insulin and HOMA-IR: *ABCG1* (cg06500161); marginally significant site also in *ABCG1* (cg1881899, *p* = 3.36 × 10^−6^), associated with HOMA-IR only. No DMS associated with fasting glucose	0 (no information about insulin levels in Lifelines)
Ronn et al, 2015 [35] ^a^	96 healthy male participants for discovery stage, 94 healthy female participants for validation stage, 2 separate EWASs performed	96 male 94 female	Cross-sectional association with age, BMI and HbA_1c_	Adipose tissue	450k	FDR < 5%, q < 0.05	Sex, family number; pedigree, age, BMI	711 DMS associated with HbA_1c_ were found in the male cohort with most significant negative correlation at *ANKRD11* gene; 7 DMS associated with HbA_1c_ were found in the female cohort, none of which were significantly associated with HbA_1c_ level in the male cohort	11 from male cohort

^a^Studies included into replication study to provide CpGs, which passed strict Bonferroni correction threshold

^b^Multiple-testing threshold calculated originally by the authors of particular study

^c^0 in last column means zero CpG sites passed study-specific Bonferroni correction threshold

C, controls; DMS, differentially methylated sites; DMR, differentially methylated region; FDR, false discovery rate; HOMA-IR, homeostatic model assessment; NASH, non-alcoholic steatohepatitis; P, patients; PC, principal component; T2D, type 2 diabetes; 27k, Infinium HumanMethylation27 BeadChip; 450k, Infinium HumanMethylation450 BeadChip

#### Study design

The majority of the reviewed EWASs (18 out of 19) used a cross-sectional design, in which phenotype and DNA methylation profile were measured at the same time point either in unrelated individuals (type 2 diabetic and healthy control participants, 15 studies) or in twin pairs, discordant for type 2 diabetes (three studies) (Tables 1, 2). Strengths of this approach typically include a large study population selected from ongoing cohorts and the possibility to adjust for existing confounders like BMI or smoking. However, a cross-sectional approach cannot establish whether the difference in methylation preceded the onset of type 2 diabetes.

### Tissue

1. Blood: The interpretation of blood-based EWASs results can be difficult, because many top hits from EWASs are known genes from immune response and inflammatory pathways, which can be mediated by the blood cell composition and, thus, do not reflect true associations with type 2 diabetes. Six out of ten blood-based studies used the reference-based estimation methods by Houseman [20] or Jaffe [37] to adjust for confounding effects of cell composition. Results from the majority of those studies indicate that differentially methylated sites in the *TXNIP*, *ABCG1*, *CPT1A* and *SREBF1* genes are associated with type 2 diabetes and glycaemic traits [21, 23–25, 33, 34].
2. Pancreas: The pancreas plays a key role in maintaining normoglycaemia through insulin secretion in response to blood glucose elevation [9]. In addition to the ten EWASs performed in blood, four of the included studies examined the association between DNA methylation in pancreas and type 2 diabetes. These studies were conducted in a limited number of individuals (*n* = 16 to 87) [27, 28] and no overlap in identified CpGs was found between the studies when considering specific multiple-testing corrections applied by the authors (FDR < 5% [12, 27]; *p* < 0.01 and 15% group-wise difference on methylation [28]). Interestingly, one study used whole-genome bisulphite sequencing (WGBS) and identified over 25,000 differentially methylated regions across the whole genome, suggesting large changes in methylome associated with type 2 diabetes [14].
3. Liver: Another important organ in glucose metabolism is the liver where, in diabetic individuals, suppression of hepatic glucose output by insulin is reduced, contributing to hyperglycaemia [38]. The exact pathophysiology causing liver insulin resistance is still unknown, suggesting a role for epigenetic mechanisms. We found two EWASs performed in liver tissue (Table 1) using rather small sample sizes (*n* = 15 [32] and 95 [31]). The majority of CpGs showing a significant methylation difference from these two studies were hypomethylated in individuals with type 2 diabetes compared with control individuals (92% and 94%, FDR < 25% and FDR < 5%, respectively). No overlap was found between liver and blood-based results of EWASs, suggesting that significant CpGs from liver EWASs might be tissue specific.
4. Adipose tissue: Pathogenesis of glucose intolerance is also associated with adipocyte metabolism and altered fat topography [39]. Among the reviewed studies, three EWASs were performed in adipose tissue: two investigating an association with type 2 diabetes (one study with five twin pairs and another with unrelated individuals, *n* = 95) and one investigating an association with HbA_1c_ level (96 healthy male, 94 healthy female participants) [29, 30, 35]. We observed no overlap (manually checked) in the top 100 CpGs from the two studies focusing on type 2 diabetes [29, 30].

#### Ethnicity

In 2013, the highest diabetes prevalence was observed in the North American and Caribbean region (around 11%), while the lowest was in the African region (around 5.7%) [40], suggesting differences in prevalence between ethnic groups. In the recent EWAS, the total risk of developing type 2 diabetes was three times higher in Indian Asians than in Europeans, regardless of differences in adiposity, physical activity and glycaemic values [21]. The authors estimated that 32% of the unexplained risk for future type 2 diabetes among Indian Asians compared with controls was associated with a higher methylation score based on the top five markers at *TXNIP*, *ABCG1*, *SREBF1*, *SOCS3* and *PHOSPHO1* [21]. A family-based study of 859 Mexican Americans showed that the degree of methylation at top regions including *TXNIP*, *ABCG1* and *SAMD12* genes and two intragenic regions accounted for 7.8% of the heritability of type 2 diabetes in Mexican Americans [25]. An EWAS performed in an Arab population showed that around 10% of methylation sites with FDR < 1% had median heritability of 0.7, supporting previous findings [22, 41]. These differences in DNA methylation between ethnic groups can be partly explained by their genetic ancestry, but also environmental and lifestyle factors may contribute to the variation, while some methylation loci (*TXNIP* or *ABCG1*) were found in populations with divergent ethnic backgrounds [21, 23–25].

### Replication study

#### Selected CpGs

From the 19 studies included in the review, we selected 15 studies (Fig. 1). A list of CpGs robustly associated with type 2 diabetes or glycaemic traits was compiled based on the application of a stringent study-specific multiple-testing correction threshold to avoid false positive results (see Methods). After the removal of duplicates, we obtained a list of 100 unique CpGs (ESM Table 1) identified in peripheral blood (52 for type 2 diabetes and 21 for fasting glucose), pancreas (15 for type 2 diabetes), adipose tissue (ten for HbA_1c_ blood level) and liver (two for type 2 diabetes).

#### Study population

We investigated which of the above-mentioned EWASs findings, both in blood and in other tissues, could be replicated in blood samples from the Lifelines case–control sample (for clinical characteristics see Table 3 and ESM Table 2). Individuals with type 2 diabetes were older, had a significantly higher BMI, waist–hip ratio and blood pressure, as well as higher levels of HbA_1c_, fasting glucose and triacylglycerols compared with control individuals. We observed no differences in socioeconomic status represented by level of education between type 2 diabetic and control participants (Table 3).

**Table 3:** Baseline characteristics of the study sample of type 2 diabetic individuals and healthy individuals from the Lifelines cohort (*n* = 198)

	Type 2 diabetic individuals (*n* = 100)	Control individuals (*n* = 98)^a^	*p* value
Sex (M) (*n*, %)	52 (52)	44 (44.9)	0.44
Age (years)	62 (53–69)	50 (46–63)	3 × 10^−8^
BMI (kg/m^2^)	30.8 ± 4.7	25.3 ± 3.6	< 2.2 × 10^−16^
Waist (cm)	105.3 ± 12.4	89.2 ± 11.0	< 2.2 × 10^−16^
Waist–hip ratio	0.98 ± 0.08	0.9 ± 0.08	1.1 × 10^−10^
Fasting status^b^	98 (98)	97 (99)	0.57
Biochemical measurements			
HbA_1c_ (%)	6.6 (6.4–8.5)	5.6 (5.3–5.7)	< 2.2 × 10^−16^
HbA_1c_ (mmol/l)	49 (45.8–55.3)	37.5 (35.3–39)	< 2.2 × 10^−16^
Fasting glucose (mmol/l)^c^	7.4 (6.4–8.5)	4.9 (4.6–5.3)	< 2.2 × 10^−16^
Triacylglycerol (mmol/l)	1.4 (1.1–1.9)	1.0 (0.7–1.2)	2.2 × 10^−8^
HDL-cholesterol (mmol/l)	1.2 ± 0.32	1.54 ± 0.4	1.6 × 10^−8^
LDL-cholesterol (mmol/l)	2.8 ± 0.9	3.5 ± 0.9	3 × 10^−7^
Total cholesterol (mmol/l)	4.5 ± 1.0	5.3 ± 1.0	1.1 × 10^−7^
Systolic BP (mmHg)	135 ± 18	122 ± 11	4.2 × 10^−9^
Diastolic BP (mmHg)	76 ± 9	73 ± 7	6.7 × 10^−3^
Education level (*n*, %)^c^			
Low Intermediate High	55 (59) 22 (24) 16 (17)	34 (37) 28 (30) 30 (33)	0.2
Insulin use (*n*, %)	10 (10)	0 (0)	–
Oral blood glucose lowering drugs (*n*, %)	51 (51)	0 (0)	–
Lipid lowering drugs (*n*, %)	60 (60)	1 (1)	–

Normal distribution assessment based on histograms and probability–probability plots

Data are shown as mean ± SD for normally distributed variables, as median and 25th and 75th quintile for not normally distributed variables and as number of individuals (%) for categorical variables

*p* values are obtained from Student’s *t* test for normally distributed variables or from Mann–Whitney *U* test for not normally distributed variables and *χ* ^2^ square for categorical variables. Significant *p* values < 0.05

^a^Two controls were excluded because of a sex mismatch (between actual data and methylation data)

^b^Fasting status data apply to all biochemical blood measurements presented in the table

^c^Fasting glucose value missing for one individual; education level missing for seven individuals

#### Association with type 2 diabetes: blood-specific CpGs

First, we analysed the 52 CpGs associated with type 2 diabetes in blood (ESM Table 1). In our Lifelines sample, five out of 52 included CpGs showed significant associations with type 2 diabetes (the Bonferroni-adjusted *p* < 0.0009 (0.05/52 CpGs)), including the loci in the *ABCG1*, *LOXL2*, *TXNIP*, *SLC1A5* and *SREBF1* genes (see a short description in ESM Box 1). This number increased to 15 CpGs when using the nominal significance level (*p* < 0.05) (Table 4). In agreement with previous studies, we observed hypermethylation in the loci at the *ABCG1* and *SREBF1* genes and hypomethylation in *TXNIP*, *LOXL2* and *SLC1A5* in type 2 diabetic compared with control individuals. Also, all nominally significant associations showed the same direction of effect as in the original EWASs. After adjustment for BMI, only the CpG site in *ABCG1* remained significantly associated with type 2 diabetes, while for all other CpGs effect sizes became smaller and were no longer significant (ESM Fig. 1). Based on β values from regression analysis, we concluded that associations between significant CpGs and type 2 diabetes are partly explained by BMI (BMI accounted for 5–70% of variance, data not shown). Additional adjustment for other factors (see Methods) demonstrated that these covariates had only a relatively small impact on effect sizes and *p* values (ESM Table 3). Furthermore, we performed a sensitivity analysis on subsamples (see Methods), in which only the CpGs in *TXNIP* (50 vs 49) and *ABCG1* (100 vs 49) reached the significance threshold (*p* < 0.0009), suggesting lack of power compared with the total group comprising 198 samples (data not shown). We also examined, for the 15 nominally significant CpGs, whether the differences in methylation were influenced by the occurrence of complications in diabetic individuals. We found no significant difference between individuals with and without complications (ESM Table 4). Finally, to check the effect of inflammation, we also adjusted the analysis for C-reactive protein (CRP) level and found no difference in the outcome (data not shown).

**Table 4:** Significant differentially methylated CpGs for type 2 diabetes as originally identified in blood and replicated in the Lifelines type 2 diabetes EWAS sample in blood (*n* = 198)

Illumina ID	CHR	MAPINFO	Gene name	Location in gene	Location in CpG island	Mean methylation (%)	Model 1		Model 1 + BMI	
							Delta methylation (%)	*p* value	Delta methylation (%)	*p* value
cg06500161^a^	21	43656587	*ABCG1*	Body	Shore	60.9	3	2.9 × 10^−7^	2.39	3 × 10^−4^
cg24531955^a^	8	23154691	*LOXL2*	3′UTR	Open sea	25.4	−1.99	1.6 × 10^−4^	−1.63	6 × 10^−3^
cg19693031	1	145441552	*TXNIP*	3′UTR	Open sea	69.5	−3.6	2.5 × 10^−4^	−2.68	1.5 × 10^−2^
cg02711608^a^	19	47287964	*SLC1A5*	1stExon	Shelf	20.1	−1.81	3.2 × 10^−4^	−1.26	2.7 × 10^−2^
cg11024682^a^	17	17730094	*SREBF1*	Body	Shelf	44.6	1.88	5.5 × 10^−4^	1.04	8 × 10^−2^
cg07960624	8	119208486	*SAMD12*	3′UTR	Open sea	39.7	−2.3	4.8 × 10^−3^	−1.59	9 × 10^−2^
cg03497652	16	4751569	*ANKS3*	Body	Open sea	55.5	1.86	9.7 × 10^−3^	1.79	3 × 10^−2^
cg19266329	1	145456128	*POLR3GL* ^b^	–	Open sea	60.9	−1.77	1 × 10^−2^	−0.98	0.20
cg22909677	6	109172312	*ARMC2*	5′UTR	Shelf	80.4	1.11	1.2 × 10^−2^	1.04	0.07
cg08309687^a^	21	35320596	*ATP5O* ^b^	–	Open sea	56.7	−2.61	1.5 × 10^−2^	−0.92	0.36
cg26804423^a^	7	8201134	*ICA1*	Body	Open sea	63.8	1.39	1.5 × 10^−2^	0.78	0.23
cg13199639	6	33360495	*KIFC1*	Body	Shore	11.7	−0.91	1.9 × 10^−2^	−0.49	0.34
cg15962267	5	138612986	*SNHG4*	Body	Shelf	69.9	−1.29	2.9 × 10^−2^	−0.79	0.22
cg03725309^a^	1	109757585	*SARS*	Body	Shore	17.9	−1.06	3.4 × 10^−2^	−0.72	0.19
cg10919522^a^	14	74227441	*C14orf43*	5′UTR	Shore	31.4	−1.42	4 × 10^−2^	−0.42	0.56

No CpGs originally identified in other tissues were replicated in the present study looking at methylation in blood

Delta methylation is based on β values; *p* values are from analyses based on M values

Abbreviations: CHR, chromosome; MAPINFO, position on the chromosome; Shore, 0–2 kb from CpG island; Shelf, 2–4 kb from CpG island; Open sea, more than 4 kb from CpG island

Significant *p* values below 9.6 × 10^−4^ based on Bonferroni calculation

^a^CpGs also found to be associated with BMI in recently published EWAS [42]

^b^Closest genes were *POLR3GL* (108 bp downstream) and *ATP5O* (32,438 bp upstream)

Next, we investigated whether the five replicated type 2 diabetes-associated CpGs are also correlated with glycaemic and lipid phenotypes of healthy individuals (*n* = 98, Table 5). The methylation level at the *ABGC1* site was significantly and positively correlated with age, fasting glucose and triacylglycerols, while the methylation levels of the *TXNIP* and *SLC1A5* CpGs was negatively correlated with age. The methylation level at *SREBF1* was positively correlated with both fasting glucose and lipid levels. No significant correlation with BMI was found in healthy individuals.

**Table 5:** Correlations between DNA methylation (β values) of five replicated CpGs with type 2 diabetes risk factors in healthy individuals in Lifelines sample (*n* = 98)

	*ABCG1*		*LOXL2*		*TXNIP*		*SLC1A5*		*SREBF1*	
	*r*	*p* value	*r*	*p* value	*r*	*p* value	*r*	*p* value	*r*	*p* value
Age	0.31	1.7 × 10^−3^	−0.17	8 × 10^−2^	−0.11	3.4 × 10^−2^	−0.27	6.6 × 10^−3^	0.45	4.4 × 10^−6^
Fasting glucose	0.31	1.9 × 10^−3^	−0.09	0.33	−0.15	0.14	−0.01	0.86	0.21	3.5 × 10^−2^
Triacylglycerol	0.25	1.3 × 10^−2^	−0.11	0.26	−0.17	9 × 10^−2^	−0.13	0.18	0.23	2.2 × 10^−2^
Total cholesterol	0.15	0.14	−0.10	0.32	−0.11	0.24	0.03	0.75	0.44	6.7 × 10^−6^
LDL-cholesterol	0.15	0.13	0.11	0.25	−0.14	0.16	−0.03	0.71	0.41	2.8 × 10^−5^
HDL-cholesterol	−0.07	0.46	0.04	0.65	0.09	0.37	0.27	6.4 × 10^−3^	0.07	0.44
BMI	0.19	0.065	−0.1	0.35	−0.12	0.26	−0.16	0.1	0.15	0.12

*r* = Pearson’s correlation coefficient

Significant *p* values < 0.05

#### Associations with type 2 diabetes: other tissue-specific CpGs

In addition to the 52 CpGs associated with type 2 diabetes in blood, we also analysed 17 CpGs that were associated with type 2 diabetes in pancreas and liver to test whether DNA methylation in metabolically active tissues may be reflected in DNA methylation in blood. No significant associations were found for any of these CpGs in blood samples (all *p* > 0.1).

#### Associations with glycaemic traits

Finally, we tested the CpGs previously shown to be associated with fasting glucose and HbA_1c_ levels. In blood samples from the 98 healthy individuals, we replicated the association between CpGs in the *CCDC57* and *ABCG1* genes and fasting glucose level at nominal significance (*p* < 0.05, Table 6). Interestingly, after adjustment for BMI, two more CpGs, located in *MDN1* and *FLAD1* genes reached nominal significance (Table 6). We found no significant association between the level of HbA_1c_ and DNA methylation at any of the ten CpGs identified in adipose tissue.

**Table 6:** Significant differentially methylated CpGs for fasting glucose replicated in healthy control individuals from the Lifelines type 2 diabetes EWAS subsample (*n* = 98)

Illumina ID	CHR	MAPINFO	Gene name	Location in gene	Location in CpG island	Mean methylation (%)	Model 1		Model 1 + BMI	
							Delta methylation (%)	*p* value	Delta methylation (%)	*p* value
cg06500161	21	43656587	*ABCG1*	Body	Shore	59.1	1.82	6.8 × 10^−3^	1.68	1.6 × 10^−3^
cg06715330	17	80158206	*CCDC57*	Body	Open sea	81.3	−1.82	0.01	−2.05	6.6 × 10^−3^
cg16809457	6	90399677	*MDN1*	Body	Open sea	56.6	1.71	0.08	2.08	0.04
cg16097041	1	154965544	*FLAD1*	3′UTR	Open sea	59.4	1.27	0.09	1.61	0.04

Delta methylation is based on β values; *p* values are based on M values

Significant *p* values < 0.05

CHR, chromosome; MAPINFO, position on the chromosome; Shore, 0–2 kb from CpG island; Shelf, 2–4 kb from CpG island; Open sea, more than 4 kb from CpG island

#### The EWASs for other metabolically relevant traits

Since high BMI and dyslipidaemia are well-known risk factors for type 2 diabetes and are commonly observed in diabetic individuals [43], we compared the results from our replication study with the results from recent EWASs studying DNA methylation related to adiposity and blood lipids [42, 44–46]. We found a large overlap between CpGs that are significantly associated with BMI and triacylglycerol levels, and those that are associated with type 2 diabetes and fasting glucose (ESM Table 5).

## Discussion

In this study, we first comprehensively reviewed recently published EWASs investigations of DNA methylation patterns associated with type 2 diabetes and glycaemic traits. The potential use of DNA methylation as biomarker for type 2 diabetes is frequently reported in the literature, mostly using cross-sectional approaches. A more ideal setting for testing biomarkers would be to capture changes in the methylation profile prior to disease onset. A longitudinal study design would allow for this, since it provides measurements of methylation at multiple time points in the same individual, thereby capturing the epigenetic dynamics during life. However, due to higher costs and study duration, such EWASs are scarce, especially for complex diseases. To date, only one longitudinal EWAS study focusing on type 2 diabetes has been published, identifying five CpGs associated with disease onset in Indian Asians during the follow-up period [21], two of which (the CpGs in *ABCG1* and *PHOSPHO1)* were replicated in a prospective study [47]. In our analysis we replicated three CpGs from the longitudinal study (i.e. *ABCG1*, *TXNIP* and *SREBF1*) indicating that those differences in methylation can also be captured in a cross-sectional study, for example, due to the stability of methylation level after disease onset. These CpGs represent potential predictive biomarkers for type 2 diabetes susceptibility.

Another issue concerns the inconsistency in EWASs methylation levels across tissues and whether blood can serve as a proxy tissue to capture these patterns. Changes in DNA methylation have been reported for different tissues like pancreas, liver, skeletal muscle or adipose tissue relevant in type 2 diabetes (ESM Table 1) [27, 31, 32, 48, 49]. The overlap in those results is limited, suggesting that the majority of the identified DNA methylation loci are tissue specific. However, some studies reported an overlap in disease-specific and age-related differentially methylated CpGs between blood and other relevant tissues. In recent EWASs, around 60% of the methylation changes associated with age in pancreatic islets also occur in blood, including *FHL2*, *KLF14*, *FAM123C* and *GNPNAT1*, all genes known to be associated with type 2 diabetes or insulin secretion [12]. Chambers et al reported that two out of five tested CpGs (in *TXNIP* and *SOCS3*) were differentially methylated in liver and reflected in blood [21]. Interestingly, another recent study showed hypermethylation at a CpG located in the *SREBF1* gene in pancreatic cells and blood from type 2 diabetic individuals, and hypomethylation at the *TXNIP* locus in pancreatic islets, skeletal muscle and blood, which is directionally consistent with our findings in blood [47]. Taken together, these data indicate that some methylation changes found in the other tissues can be mirrored in blood. However, in our study we did not replicated the CpGs from the liver, pancreas and adipose tissue EWASs. This may be due to the small discovery sample sizes, the relatively small sample size of our replication study and/or reflect tissue-specific methylation patterns.

Epigenetic changes can be either a cause or a consequence of disease or an indirect contributing factor through environmental exposures that can affect both epigenome and type 2 diabetes risk [50]. Multiple factors can affect DNA methylation, such as environmental exposures [51], psychosocial [52] and genetic factors [53], together explaining the variance in DNA methylation levels between individuals. Also, accumulating data indicate that interactions between genetics and epigenetics influence gene expression levels in relevant metabolic traits, leading to the development of complex diseases [54, 55]. Recently, genetic ancestry and ethnicity is also shown to influence the methylation level [41]. Between the EWASs reviewed, we observed an overlap for a number of CpGs (*TXNIP*, *ABCG1*, *SOCS3*, *SREBF1* and *CPT1A)* from EWASs performed in blood samples from Europeans, Indian Asians, Mexican Americans and Arabs, suggesting an association of DNA methylation with type 2 diabetes at these sites, irrespective of ethnic, social and environmental differences. Moreover, this finding highlights the usefulness of data sharing to create opportunities to perform meta-analyses, as is common practice for genome-wide association studies (GWASs).

In this study, we replicated five CpGs in blood, from which four reside in the genes previously shown to be associated with type 2 diabetes (*ABCG1*, *LOXL2*, *SLC1A5*, *SREBF1*) (ESM Box 1). Another replicated CpG site is *TXNIP* (cg19693031), which is shown to be hypomethylated in type 2 diabetes [21, 23–25]. Expression of *TXNIP* has been linked to glucose levels (ESM Box 1). Despite its important function in type 2 diabetes pathogenesis, *TXNIP* was not identified as one of the susceptibility genes in recent GWAS studies for type 2 diabetes [6]. These data suggest that DNA methylation is the major mechanism of controlling *TXNIP* expression, thereby affecting glucose homeostasis.

Blood cell composition can influence EWAS analyses and outcomes. There are several ways to avoid potential confounding effects of the cell composition, such as adjustment for direct measured cell count or reference-based cell count (e.g. the Houseman method [20]). In our analysis we observed no difference in effect sizes for the CpGs showing a significant association when using either the Houseman method or the measured cell count approach for adjustment, suggesting that these two methods may be used interchangeably (data not shown). Especially in studies in which information on blood cell composition is not available, methods such as the Houseman approach are essential.

It has been recently shown that methylation changes of the CpGs located in *SREBF1*, *ABCG1* and *CPTA1* were not only associated with type 2 diabetes but also with BMI [42, 44, 46]. Therefore, we compared our results with those from recent EWASs for adiposity and other relevant metabolic phenotypes [42, 44, 46]. We observed a substantial overlap between BMI and triacylglycerol-related CpGs, and CpGs associated with type 2 diabetes and glycaemic traits. Approximately 60% to 70% of diabetic individuals show some lipid abnormalities, which are associated with insulin resistance. The observed overlap in EWASs results could be explained by the fact that hypertriacylglycerolaemia leads to elevated non-esterified fatty acid levels, which in turn could induce insulin resistance and beta cell dysfunction [56]. Next, recent findings from the EWASs for adiposity indicate that adiposity determines methylation level at the majority of the identified loci [42] and that the methylation changes in blood might in part be a consequence of the alterations in lipid and glucose metabolism associated with BMI. In this EWAS, 62 of the 187 BMI methylation loci were associated with incidence of type 2 diabetes, and the BMI methylation risk score, calculated based on those CpGs, predicted future development of type 2 diabetes [42]. Together, this supports the hypothesis that BMI accounts partly for the association between DNA methylation and type 2 diabetes.

Overall, we conclude that a number of differentially methylated CpGs associated with type 2 diabetes in the published EWASs can be replicated in blood and show promise as disease biomarkers. Our data indicate that BMI partly explains the associations between DNA methylation and type 2 diabetes (i.e. only five out of 15 CpGs remained significant after adjustment for BMI). Whether these markers can be used as biomarkers for type 2 diabetes in a clinical practice requires further investigation. We recommend that more longitudinal studies are performed to confirm the robustness of these markers and to identify additional potential markers.

## Electronic supplementary material

ESM (PDF 840 kb)

## Abbreviations

CpG: Cytosine-phosphate-guanine
CVD: Cardiovascular disease
EWAS: Epigenome-wide association study
GWAS: Genome-wide association study
WGBS: Whole-genome bisulphite sequencing
