# Supplementary material for: DNA methylation markers associated with type 2 diabetes, fasting glucose and HbA1c levels: a systematic review and replication in a case–control sample of the Lifelines study
Source: Diabetologia. 2017 Nov 21;61(2):354–68. doi: 10.1007/s00125-017-4497-7 (PMC6448925; doi:10.1007/s00125-017-4497-7)
Supplement: Supplementary file 1 — (PDF 840 kb) [file 125_2017_4497_MOESM1_ESM.pdf]

Content

ESM Methods 2

ESM Box 1 4

ESM Table 1 5

ESM Table 2 9

ESM Table 3 10

ESM Table 4 11

ESM Table 5 12

ESM Figure 1 13

ESM Methods

Search terms

Pubmed database was searched using the following search terms: (("Diabetes Mellitus, Type 2"[MeSH] OR (type 2 diabete[tiab] OR type 2 diabetes[tiab] OR type 2 diabetes,[tiab] OR type 2 diabetic[tiab] OR type 2 diabetics[tiab]) OR "hemoglobin A, Glycosylated"[MeSH] OR hbac1[tiab] OR fasting glucose[tiab]) AND ("DNA Methylation"[MeSH] OR DNA methylation[tiab] OR EWAS[tiab] OR Epigenome-wide association study[tiab] OR methylome[tiab])) NOT ("animals"[MeSH Terms] NOT "humans"[MeSH Terms]) The Embase database was searched using: (((('non insulin dependent diabetes mellitus'/exp OR 'hemoglobin a1c'/exp OR 'fasting glucose') AND ('dna methylation'/exp OR 'dna methylation assay'/exp OR 'ewas' OR 'epigenome wide association study')) NOT ([animals]/lim NOT [humans]/lim)) restricted to articles only (not reviews, letter, comments, conference posters). Both searches were performed until April 26, 2017. Of each publication, both title and abstract were examined to evaluate its usefulness for review by two independent reviewers (EW; JvVO). We identified 580 relevant publications in PubMed and Embase. EWASs investigating DNA methylation patterns associated with T2D or glycaemic traits were included. We excluded candidate-gene approach studies, studies that focused on epigenetic mechanisms other than DNA methylation, studies determining only global methylation, studies not related to T2D or glucose metabolism, intervention studies, animal studies, studies in neonates, case reports, promoter methylation, EWAS replication studies, co-morbidities (e.g. dementia in elderly with T2D, Parkinson, Alzheimer), T2D complications (e.g. nephropathy, patient-derived diabetic foot ulcer fibroblasts) and articles published in other languages than English. Disagreements between the two reviewers were solved through discussion and with help of an arbitrator (HS). Full texts of included studies were retrieved.

Quality control and normalization

Methylumi R package was used to extract the data from the raw IDAT files. We performed quality control checks on the probes and samples. Two samples were excluded from the control group for sex-mismatch. After having performed a principal component analysis (PCA), we checked for outliers using the first two principal components (PCs). Next, we performed background correction and probe type normalization based on the Touleimat and Tost pipeline[1], using the dasen normalization strategy[2]. We remapped the 450K probes to the human genome reference (HG19). Details on this procedure can be found in Bonder et al [3]. Next, we removed probes with a known Single Nucleotide Polymorphism (SNP) from “Genome of the Netherlands” (GoNL), minor allele frequency (MAF) > 0.01) at the single base extension (SBE) site or CpG site. Lastly, we removed

all probes on the sex chromosomes, resulting in 423,289 high quality methylation sites for subsequent analyses.

Assessing the quality of included publications

We evaluated bias and quality of included publications using the Newcastle-Ottawa Scale, a scale designed to evaluate the quality of case-control and cohort studies [4]. Two independent reviewers (EW; JvVO) assessed the study quality based on the three categories: selection of individuals, comparability of cases and controls and outcome assessment. A maximum of nine stars could be granted. Studies with a score of eight or nine stars were judged to be at low risk of bias, studies with a score of six to seven stars were considered to be at medium risk of bias, whereas studies with a score of five or less stars were considered to be at high risk of bias.

ESM Box 1 Functions of 5 top CpG sites from our replication study in Lifelines T2D EWAS sub study.

ABCG1 is involved in lipid homeostasis, especially promoting cholesterol efflux into HDL[5]. Cholesterol homeostasis is known to be essential modulator of insulin secretion, which is reflected in the phenotype of the *ABCG1*-/- mice, characterized by impaired glucose tolerance and insulin secretion[6].
LOXL2 gene, a member of lysyl oxidase family and an important regulator of tumour progression[7]. It has been shown that *LOXL2* expression is glucose-sensitive in eye and can be related to diabetic retinopathy[8]. In mice, *LOXL2* inhibitor has a profitable effect on glomerular structure in kidney, implicating that *LOXL2* can be a potential therapeutic target for diabetic nephropathy[9].
TXNIP (Thioredoxin-interacting protein) expression is induced by glucose in β-cells as well as in other tissues and also inhibits glucose uptake via enhanced endocytosis of glucose-transporter 1 (GLUT1)[10].
SREBF1 gene located on chromosome 17 encodes two protein - SREBP-1a and SREBP-1c, both participating in the synthesis of cholesterol, fatty acids and triglycerides. In liver overexpressed SREBP-1c induces the secretion of glucokinase, an enzyme involved in glucose utilization. Moreover, in obese mice with insulin resistance and hyperinsulinemia, SREBP-1c levels are elevated[11]
SLC1A5 gene- its protein belongs to different solute carriers (SLC) family of amino-acids transporters. Overexpression of *SLC1A5* increases glutamine uptake especially in cancer cells, therefore represents a potential target in cancer as well as in pathologies such as obesity, type 2 diabetes and neurodegeneration[12]

ESM Table 1 List of CpG sites associated with T2D and glycaemic traits used for replication analysis (100 unique CpGs).

CpG site	Gene name	Study, year	Effect sizes		p-values	Tissue	Correction	Trait
			β	OR				
**cg19693031**	TXNIP	Chamber et. al, 2015		RR=0.92	$1x10^{-13}$	Blood	Bonferroni	T2D
**cg19693031**	TXNIP	Sorriano-Tarraga et. al, 2016	-0.13		$7.3x10^{-16}$	Blood	Bonferroni	T2D
**cg19693031**	TXNIP	Florath et. Al, 2016	-0.03		$4.5x10^{-7}$	Blood	Bonferroni	T2D
**cg19693031**	TXNIP	Kulkarni et.al, 2015*	-0.58		$1.53x10^{-19}$	Blood	Bonferroni	T2D
**cg06500161**	ABCG1	Kulkarni et.al, 2015	0.39		$9.43x10^{-10}$	Blood	Bonferroni	T2D
**cg06500161**	ABCG1	Chamber et. al, 2015		RR=1.08	$2.2x10^{-13}$	Blood	Bonferroni	T2D
**cg11024682**	SREBF1	Chamber et. al, 2015		RR=1.06	8.4×10^{-9}	Blood	Bonferroni	T2D
**cg06721411**	DOX1	Al Muftah et al. 2016	5.79		$1.18x10^{-9}$	Blood	Bonferroni	T2D
**cg02650017**	PHOSPHO1	Chamber et. al, 2015		RR=0.94	$2.1x10^{-9}$	Blood	Bonferroni	T2D
**cg00076653**	C1QTNF7	Kulkarni et.al, 2015	-0.4		$6.38x10^{-9}$	Blood	Bonferroni	T2D
**cg00277397**	CALN1	Kulkarni et.al, 2015	0.43		$3.4x10^{-8}$	Blood	Bonferroni	T2D
**cg00574958**	CPT1A	Kulkarni et.al, 2015	0.38		$4.29x10^{-9}$	Blood	Bonferroni	T2D
**cg01657995**	C6orf48;SNORD52	Kulkarni et.al, 2015	0.48		$9.3x10^{-8}$	Blood	Bonferroni	T2D
**cg18181703**	SOCS3	Chamber et. al, 2015		RR=0.95	$2.1x10^{-7}$	Blood	Bonferroni	T2D
**cg02560388**		Kulkarni et.al, 2015	0.46		$5.85x10^{-8}$	Blood	Bonferroni	T2D
**cg02711608**	SLC1A5	Kulkarni et.al, 2015	0.45		$4.17x10^{-10}$	Blood	Bonferroni	T2D
**cg03497652**	ANKS3	Kulkarni et.al, 2015	-0.41		$2.61x10^{-8}$	Blood	Bonferroni	T2D
**cg03699074**	FAM38A	Kulkarni et.al, 2015	0.39		$1.1x10^{-8}$	Blood	Bonferroni	T2D
**cg03725309**	SARS	Kulkarni et.al, 2015	0.73		$4.68x10^{-11}$	Blood	Bonferroni	T2D
**cg04344749**	LDLRAP1	Kulkarni et.al, 2015	-0.35		$1x10^{-7}$	Blood	Bonferroni	T2D
**cg04645070**		Kulkarni et.al, 2015	0.70		$6.08x10^{-9}$	Blood	Bonferroni	T2D
**cg04727071**	ZBTB7A	Kulkarni et.al, 2015	0.60		$3.45x10^{-8}$	Blood	Bonferroni	T2D
**cg04816311**	C7orf50	Kulkarni et.al, 2015	-0.50		$5.47x10^{-8}$	Blood	Bonferroni	T2D
**cg04973995**		Kulkarni et.al, 2015	0.45		$4.32x10^{-8}$	Blood	Bonferroni	T2D
**cg04992150**	NUP210	Kulkarni et.al, 2015	0.60		$1.1x10^{-7}$	Blood	Bonferroni	T2D
**cg05400498**		Kulkarni et.al, 2015	-0.46		$1.12x10^{-9}$	Blood	Bonferroni	T2D

**cg06007201**	FAM38A	Kulkarni et.al, 2015	0.49	$2.73x10^{-8}$	Blood	Bonferroni	T2D
**cg06178887**	POU2F2	Kulkarni et.al, 2015	-0.51	$3.26x10^{-8}$	Blood	Bonferroni	T2D
**cg07092212**	DGKZ	Kulkarni et.al, 2015	0.67	$9.52x10^{-8}$	Blood	Bonferroni	T2D
**cg07960624**	SAMD12	Kulkarni et.al, 2015	0.45	$1.55x10^{-9}$	Blood	Bonferroni	T2D
**cg08309687**		Kulkarni et.al, 2015	0.47	$2.45x10^{-9}$	Blood	Bonferroni	T2D
**cg08788930**	DENND3	Kulkarni et.al, 2015	-0.35	$4.76x10^{-8}$	Blood	Bonferroni	T2D
**cg09247619**	PTPRC	Kulkarni et.al, 2015	0.66	$1.85x10^{-8}$	Blood	Bonferroni	T2D
**cg10508317**	SOCS3	Kulkarni et.al, 2015	0.36	$7.42x10^{-9}$	Blood	Bonferroni	T2D
**cg10919522**	C14orf43	Kulkarni et.al, 2015	0.46	$5x10^{-10}$	Blood	Bonferroni	T2D
**cg13199639**	KIFC1	Kulkarni et.al, 2015	0.38	$2.95x10^{-8}$	Blood	Bonferroni	T2D
**cg13640297**	WDR27	Kulkarni et.al, 2015	-0.71	$6.66x10^{-8}$	Blood	Bonferroni	T2D
**cg14204586**	ARHGEF2	Kulkarni et.al, 2015	0.63	$1.16x10^{-8}$	Blood	Bonferroni	T2D
**cg14597545**	ADPGK	Kulkarni et.al, 2015	-0.69	$1.5x10^{-8}$	Blood	Bonferroni	T2D
**cg15585213**	PRDM2	Kulkarni et.al, 2015	-0.32	$2.57x10^{-8}$	Blood	Bonferroni	T2D
**cg15962267**	SNHG4;SNORA74A; MATR3	Kulkarni et.al, 2015	0.55	$3.09x10^{-8}$	Blood	Bonferroni	T2D
**cg16809457**	MDN1	Kulkarni et.al, 2015	-0.48	$6.01x10^{-9}$	Blood	Bonferroni	T2D
**cg17058475**	CPT1A	Kulkarni et.al, 2015	0.43	$3.89x10^{-9}$	Blood	Bonferroni	T2D
**cg17315426**	CDH24	Kulkarni et.al, 2015	0.56	$6.52x10^{-8}$	Blood	Bonferroni	T2D
**cg17666418**		Kulkarni et.al, 2015	0.61	$6.31x10^{-8}$	Blood	Bonferroni	T2D
**cg19266329**		Kulkarni et.al, 2015	0.50	$9.98x10^{-11}$	Blood	Bonferroni	T2D
**cg21699330**	NFE2L3	Kulkarni et.al, 2015	0.47	$8.56x10^{-8}$	Blood	Bonferroni	T2D
**cg21766592**	SLC1A5	Kulkarni et.al, 2015	0.53	$1.97x10^{-9}$	Blood	Bonferroni	T2D
**cg22909677**	ARMC2	Kulkarni et.al, 2015	-0.38	$1.84x10^{-9}$	Blood	Bonferroni	T2D
**cg24531955**	LOXL2	Kulkarni et.al, 2015	0.37	$9.29x10^{-9}$	Blood	Bonferroni	T2D
**cg26546155**	RSPRY1	Kulkarni et.al, 2015	-0.57	$5.92x10^{-8}$	Blood	Bonferroni	T2D
**cg26712428**	CALHM1	Kulkarni et.al, 2015	-0.39	$2.71x10^{-8}$	Blood	Bonferroni	T2D
**cg26804423**	ICA1	Kulkarni et.al, 2015	-0.56	$5.76x10^{-9}$	Blood	Bonferroni	T2D
**cg26836479**	DEDD2	Kulkarni et.al, 2015	0.56	$1.05x10^{-7}$	Blood	Bonferroni	T2D
**cg13725826**		Dayeh et.al, 2014	Delta(%) -5.24	$1x10^{-7}$	Pancreatic islets	Bonferroni	T2D

**cg15599668**	SYNPO	Dayeh et.al, 2014	-10.45	2.9x10^{-8}	Pancreatic islets	Bonferroni	T2D
**cg22610620**		Dayeh et.al, 2014	-10.26	7.1x10^{-8}	Pancreatic islets	Bonferroni	T2D
**cg24327307**	SSBP3	Dayeh et.al, 2014	-9.14	7.7x10^{-8}	Pancreatic islets	Bonferroni	T2D
**cg19296305**	LEF1	Dayeh et.al, 2014	-9.14	1.1x10^{-8}	Pancreatic islets	Bonferroni	T2D
**cg16782117**		Dayeh et.al, 2014	-8.45	6.6x10^{-8}	Pancreatic islets	Bonferroni	T2D
**cg01649611**	THADA;THADA	Dayeh et.al, 2014	-7.81	5.4x10^{-8}	Pancreatic islets	Bonferroni	T2D
**cg15275625**		Dayeh et.al, 2014	-7.46	1.8x10^{-8}	Pancreatic islets	Bonferroni	T2D
**cg17328407**		Dayeh et.al, 2014	-7.13	8.7x10^{-8}	Pancreatic islets	Bonferroni	T2D
**cg09225373**		Dayeh et.al, 2014	-6.86	4.3x10^{-8}	Pancreatic islets	Bonferroni	T2D
**cg07820189**		Dayeh et.al, 2014	-6.59	3.2x10^{-8}	Pancreatic islets	Bonferroni	T2D
**cg22378252**	GCNT2	Dayeh et.al, 2014	-6.38	6.2x10^{-8}	Pancreatic islets	Bonferroni	T2D
**cg21449597**		Dayeh et.al, 2014	-6.18	9.7x10^{-8}	Pancreatic islets	Bonferroni	T2D
**cg04071398**	IL20RA	Dayeh et.al, 2014	-5.99	5.4x10^{-8}	Pancreatic islets	Bonferroni	T2D
**cg12458003**	NFASC	Dayeh et.al, 2014	-5.91	3.7x10^{-8}	Pancreatic islets	Bonferroni	T2D
**cg08125242**		Nilsson et. al, 2015	-0,53	1,04x10^{-9}	liver	Bonferroni	T2D
**cg07088414**		Nilsson et. al, 2015	-0,50	5,83x10^{-8}	liver	Bonferroni	T2D
**cg27402634**		Nilsson et. al, 2015	-0,77	8,08x10^{-8}	liver	Bonferroni	T2D
**cg00574958**	CPT1A	Kriebel et al., 2016	-0.10	9.8x10^{-8}	Blood	Bonferroni	Fasting glucose
**cg06500161**	ABCG1	Kriebel et al., 2016	0.04	4.2x10^{-10}	Blood	Bonferroni	Fasting glucose
**cg11024682**	SREBF1	Kriebel et al., 2016	0.06	2.5x10^{-10}	Blood	Bonferroni	Fasting glucose
**cg06500161**	ABCG1	Kulkarni et al., 2015	0.20	3.7x10^{-9}	Blood	Bonferroni	Fasting glucose
**cg00138407**	KLHL18	Kulkarni et al., 2015	0.27	4.44e^{-08}	Blood	Bonferroni	Fasting glucose
**cg00552753**		Kulkarni et al., 2015	0.20	7.97x10^{-8}	Blood	Bonferroni	Fasting glucose
**cg01676795**	POR	Kulkarni et al., 2015	0.31	2.94x10^{-8}	Blood	Bonferroni	Fasting glucose
**cg02059849**	PTP4A3	Kulkarni et al., 2015	0.21	5.91x10^{-8}	Blood	Bonferroni	Fasting glucose
**cg02079413**	SNORA54;NAP1L4	Kulkarni et al., 2015	0.22	1.94x10^{-9}	Blood	Bonferroni	Fasting glucose
**cg02436098**	TNFAIP8L1	Kulkarni et al., 2015	-0.19	4.03x10^{-8}	Blood	Bonferroni	Fasting glucose
**cg04816311**	C7orf50	Kulkarni et al., 2015	0.29	4.61x10^{-9}	Blood	Bonferroni	Fasting glucose
**cg06715330**	CCDC57	Kulkarni et al., 2015	0.19	7.65x10^{-8}	Blood	Bonferroni	Fasting glucose
**cg07136133**	PRR5L	Kulkarni et al., 2015	-0.25	4.77x10^{-10}	Blood	Bonferroni	Fasting glucose

**cg08309687**		Kulkarni et al., 2015	-0.24	$1.5x10^{-8}$	Blood	Bonferroni	Fasting glucose
**cg11467506**		Kulkarni et al., 2015	-0.27	$1.34x10^{-8}$	Blood	Bonferroni	Fasting glucose
**cg16097041**	FLAD1;LENEP	Kulkarni et al., 2015	0.27	$4.57x10^{-8}$	Blood	Bonferroni	Fasting glucose
**cg16809457**	MDN1	Kulkarni et al., 2015	0.25	$4.98x10^{-8}$	Blood	Bonferroni	Fasting glucose
**cg19048360**	PFDN6;WDR46	Kulkarni et al., 2015	-0.19	$9.05x10^{-8}$	Blood	Bonferroni	Fasting glucose
**cg19693031**	TXNIP	Kulkarni et al., 2015	-0.27	$1.55x10^{-15}$	Blood	Bonferroni	Fasting glucose
**cg22887911**	TMEM42	Kulkarni et al., 2015	-0.18	$9.85x10^{-8}$	Blood	Bonferroni	Fasting glucose
**cg23906191**	C7orf50	Kulkarni et al., 2015	0.20	$3.51x10^{-8}$	Blood	Bonferroni	Fasting glucose
**cg25217710**		Kulkarni et al., 2015	0.31	$8.88x10^{-11}$	Blood	Bonferroni	Fasting glucose
**cg26666886**	ANKRD11	Ronn et. al, 2015	-0,05	$2,3x10^{-9}$	Adipose tissue	Bonferroni	HbA1c
**cg07973479**	*ATP8A2*	Ronn et. al, 2015	-0,06	$3,1x10^{-8}$	Adipose tissue	Bonferroni	HbA1c
**cg08242859**	*IMPDH1*	Ronn et. al, 2015	-0,04	$2,8x10^{-8}$	Adipose tissue	Bonferroni	HbA1c
**cg09308803**	*LOC256880;H2AFZ*	Ronn et. al, 2015	-0,03	$2,1x10^{-8}$	Adipose tissue	Bonferroni	HbA1c
**cg14178899**	*ERRFI1*	Ronn et. al, 2015	-0,06	$1,4x10^{-8}$	Adipose tissue	Bonferroni	HbA1c
**cg18083764**		Ronn et. al, 2015	0,05	$3,4x10^{-8}$	Adipose tissue	Bonferroni	HbA1c
**cg27405128**		Ronn et. al, 2015	-0,05	$1,8x10^{-8}$	Adipose tissue	Bonferroni	HbA1c
**cg01722584**	*LOC643406*	Ronn et. al, 2015	-0,05	$4,8x10^{-8}$	Adipose tissue	Bonferroni	HbA1c
**cg18161036**		Ronn et. al, 2015	-0,04	$5,1x10^{-8}$	Adipose tissue	Bonferroni	HbA1c
**cg08146372**		Ronn et. al, 2015	-0,03	$8,0x10^{-8}$	Adipose tissue	Bonferroni	HbA1c
**cg11111226**	*SH3TC1*	Ronn et. al, 2015	-0,08	$9,7x10^{-8}$	Adipose tissue	Bonferroni	HbA1c

*Kulkarni et al: For discrete traits SOLAR returns regression coefficients that are inverted in direction. Hence, for T2D, negative regression coefficient indicates increased risk; Different colours mark the same CpG sites (duplicates).

ESM Table 2 Baseline characteristics of the study sample of T2D patients and healthy controls from Lifelines, divided into four groups.

	T2D patients with complications (n=50)	T2D patients without complications (n=50)	Age, sex matched to T2D patients controls (n=49)	Second control group (n=49)
**Sex (M) (n, %)**	29 (58%)	23 (46%)	27 (55.1%)	17 (34.7%)
**Age (years)**	65 (52-71)	62 (53-68)	60 (49-65)	48 (40-51)
**BMI (kg/m^2)**	31.5 (5.8)	30.1 (3.0)	28 (3.4)	22.6 (1.6)
**Waist (cm)**	106.7 (14.7)	103.9 (9.5)	97 (8.5)	81.5 (7.5)
**Waist –Hip ratio**	0.99 (0.08)	0.98 (0.07)	0.94 (0.07)	0.87 (0.07)
**HbA1c (%)**	6.9 (6.3-7.3)	6.6 (6.3-7.1)	5.6 (5.4-5.7)	5.5 (5.3-5.7)
**Fasting glucose (mmol/L)***	7.5 ()	7.1 (6.2-8.4)	5 (4.7-5.3)	4.8 (4.5-5.2)
**Triglycerides (mmol/L)**	1.4 (1.1-1.9)	1.4 (1.1-1.9)	1.1 (0.8-1.5)	0.9 (0.7-1.1)
**HDL cholesterol (mmol/L)**	1.21 (0.3)	1.26 (0.3)	1.4 (0.3)	1.67 (0.4)
**LDL cholesterol (mmol/L)**	2.6 (0.8)	3.0 (1.0)	3.7 (0.9)	3.2 (0.8)
**Total cholesterol (mmol/L)**	4.3 (0.8)	4.7 (1.1)	5.5 (1.0)	5.1 (1.0)
**SBP (mm Hg)**	137 (19)	133 (17)	124 (8)	121 (14)
**DBP (mm Hg)**	74 (9)	77 (9)	74 (7)	71 (8)
**Education level (n, %)***				
**1- Low**	1-29 (58%)	1- 26 (55%)	1-21 (46%)	1-13 (28%)
**2- Intermediate**	2-11 (22%)	2- 11 (23%)	2-11 (24%)	2-17 (36%)
**3- High**	3- 10 (20%)	3 -10 (22%)	3-13 (30%)	3-17 (36%)
**Insulin intake (n, %)**	9 (18%)	1 (2%)	0 (0%)	0 (0%)
**Oral anti-glycaemic drugs (n, %)**	26 (52%)	25 (50%)	0 (0%)	0 (0%)
**Lipids lowering drugs (n,%)**	36 (72%)	24 (48%)	1 (2%)	0 (0%)

ESM Table 3 Significant differentially methylated CpG sites for T2D replicated in the Lifelines T2D EWAS subsample (n=198) – additional models.

**CpG site**	**CHR**	**MAPINFO**	**Gene name**	**Mean methylation (%)**	**Model 1**		**Model 1 + BMI**		**Model 1+ newly diagnosed + medication use**		**Model 1 +Education + Smoking**		**Model 1 + complications**		**Model 1 + BMI + Education**	
					**Delta (%)**	***p*-value**	**Delta (%)**	***p*-value**	**Delta (%)**	***p*-value**	**Delta (%)**	***p*-value**	**Delta (%)**	***p*-value**	**Delta (%)**	***p-value***
**cg06500161**	21	43656587	ABCG1	60.9	3	**$2{,}9\times10^{-7}$**	2.39	**3×10^{-4}**	2.55	2.3×10^{-3}	3.08	**1.2×10^{-6}**	2.56	**1×10^{-4}**	2.4	0.1
**cg24531955**	8	23154691	LOXL2	25.4	-1.99	**1.6×10^{-4}**	-1.63	6×10^{-3}	-1.91	3.8×10^{-3}	-1.79	1×10^{-3}	-1.92	1.2×10^{-3}	-1.6	0.03
**cg19693031**	1	145441552	TXNIP	69.5	-3.6	**2.6×10^{-4}**	-2.68	0.02	-3.70	0.07	-2.49	5×10^{-3}	-3.70	7×10^{-3}	-2	0.3
**cg02711608**	19	47287964	SLC1A5	20.1	-1.81	**3.2×10^{-4}**	-1.26	0.03	-1.93	1.6×10^{-3}	-1.92	3×10^{-3}	-1.93	8×10^{-3}	-1.3	0.07
**cg11024682**	17	17730094	SREBF1	44.6	1.88	**5.5×10^{-4}**	1.04	0.08	1.80	0.18	1.83	2×10^{-3}	1.8	3×10^{-3}	1	0.36
**cg07960624**	8	119208486	SAMD12	39.7	-2.3	4.8×10^{-3}	-1.59	0.09	-2.86	0.04	-2.49	4×10^{-3}	-2.87	1.6×10^{-3}	-1.9	0.22
**cg03497652**	16	4751569	ANKS3	55.5	1.86	9.7×10^{-3}	1.79	0.03	1.55	0.23	1.59	0.03	1.55	0.05	1.8	0.17
**cg19266329**	1	145456128	POLR3GL	60.9	-1.77	0,01	-0.98	0.20	-1.85	5×10^{-3}	-1.98	8×10^{-3}	-1.85	0.02	-1.1	0.32
**cg22909677**	6	109172312	ARMC2	80.4	1.11	0,01	1.04	0.07	1.25	0.14	1.27	5×10^{-3}	1.25	0.01	1.1	0.01
**cg08309687**	21	35320596	ATP5O	56.7	-2.61	0,01	-0.92	0.36	-3.32	0.13	-2.69	0.02	-3.32	6×10^{-3}	-1.3	0.67
**cg26804423**	7	8201134	ICA1	63.8	1.39	0,02	0.78	0.23	1.15	0.11	1.47	0.02	1.15	0.08	0.9	0.38
**cg13199639**	6	33360495	KIFC1	11.7	-0.91	0,02	-0.49	0.34	-0.9	0.24	-0.93	0.03	-0.9	0.04	-0.5	0.87
**cg15962267**	5	138612986	SNHG4	69.9	-1.29	0,03	-0.79	0.22	-1.42	0.42	-1.5	0.02	-1.42	0.03	-1	0.95
**cg03725309**	1	109757585	SARS	17.9	-1.06	0,03	-0.72	0.19	-0.98	0.42	-1.4	0.01	-0.99	0.1	-0.9	0.64
**cg10919522**	14	74227441	C14orf43	31.4	-1.42	0,04	-0.42	0.56	-1.27	0.04	-1.39	0.06	-1.27	0.1	-0.7	0.77

Bonferroni threshold: 0.05/52= 9.6×10^{-4} (in bold); delta methylation is based on beta-values, *p*-values are from analyses based on M-values
*Closest gene was *POLR3GL* (108bp downstream) and ATP5O (32438bp upstream)
Model 1 is adjusted for age, sex, measured blood cell composition, position on the plate and position on the chip
Abbreviations: CHR- chromosome; IllmID- Illumina ID; MAPINFO- position on the chromosome; Shores - 0-2 kb from CpG island; Shelves - 2-4 kb from CpG island; Open sea- more than 4kb from CpG island

ESM Table 4 Methylation difference between diabetic patients with and without history of CVD from the Lifelines sample.

CpG site	CHR	MAPINFO	Gene name	Mean methylation patients without complications (%)	Mean methylation patients with complications (%)	*p*-value
cg06500161	21	43656587	*ABCG1*	62.3	63.7	0.059
cg24531955	8	23154691	*LOXL2*	24.2	24.5	0.63
cg19693031	1	145441552	*TXNIP*	67.2	67.7	0.75
cg02711608	19	47287964	*SLC1A5*	19.1	19.0	0.91
cg11024682	17	17730094	*SREBF1*	46.0	46.2	0.81
cg07960624	8	119208486	*SAMD12*	37.6	38.1	0.63
cg03497652	16	4751569	*ANKS3*	56.6	57.1	0.54
cg19266329	1	145456128	*POLR3GL*	59.6	60.4	0.30
cg22909677	6	109172312	*ARMC2*	81.1	80.4	0.21
cg08309687	21	35320596	*ATP5O*	54.2	56.5	0.1
cg26804423	7	8201134	*ICA1*	64.5	64.2	0.7
cg13199639	6	33360495	*KIFC1*	11.4	11.3	0.79
cg15962267	5	138612986	*SNHG4*	68.8	69.8	0.25
cg03725309	1	109757585	*SARS*	17.3	16.3	0.07
cg10919522	14	74227441	*C14orf43*	30.3	30.4	0.98

ESM Table 5 Overlap between CpGs that are significantly associated with T2D and fasting glucose (from Table S1) and CpGs significantly associated with lipids and BMI.

CpG site	Gene name	Trait	BMI	TG		HDL		LDL	
			Wahl	Dekker	Pfeifer	Dekker	Pfeifer	Dekker	Pfeifer
cg19693031	*TXNIP*	T2D, fasting glucose	✓	✓	✓				
cg06500161	*ABCG1*	T2D, fasting glucose	✓	✓	✓	✓	✓		
cg11024682	*SREBF1*	T2D, fasting glucose	✓	✓	✓				
cg02650017	*PHOSPHO1*	T2D	✓						
cg00574958	*CPT1A*	T2D, fasting glucose	✓	✓	✓				
cg18181703	*SOCS3*	T2D	✓						
cg02560388	*LPIN1*	T2D	✓						
cg02711608	*SLC1A5*	T2D	✓	✓					
cg03725309	*SARS*	T2D	✓						
cg10919522	*C14orf43*	T2D	✓						
cg17058475	*CPT1A*	T2D		✓					
cg24531955	*LOXL2*	T2D	✓						
cg26804423	*ICA1*	T2D	✓						
cg07136133	*PRR5L*	Fasting glucose	✓						
cg08309687		Fasting glucose	✓						

The comparison was made based on published studies[13-15]. Green check marks indicate a directionally consistent overlap.

ESM Figure 1 Correlation between effect sizes based on baseline model 1 and model 2 (Model 1 +BMI).

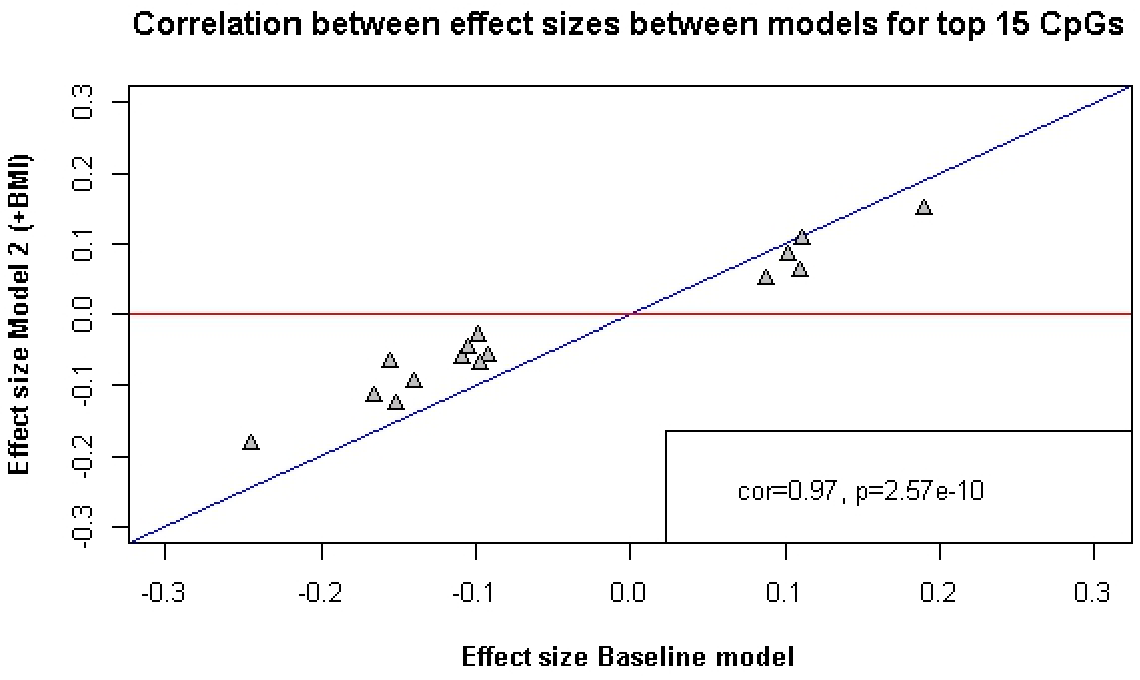

References

[1] Touleimat N, Tost J (2012) Complete pipeline for Infinium((R)) Human Methylation 450K BeadChip data processing using subset quantile normalization for accurate DNA methylation estimation. Epigenomics 4:325-341

[2] Pidsley R, Y Wong CC, Volta M, Lunnon K, Mill J, Schalkwyk LC (2013) A data-driven approach to preprocessing Illumina 450K methylation array data. BMC Genomics 14:293-2164-14-293

[3] Bonder MJ, Kasela S, Kals M, et al. (2014) Genetic and epigenetic regulation of gene expression in fetal and adult human livers. BMC Genomics 15:860-2164-15-860

[4] Wells, GA. Shea, B. O'Connell, D. Peterson, D. Welch, V. Losos, M. Tugwell, P. (2000) The Newcastle-Ottawa Scale (NOS) for assessing the quality of nonrandomised studies in meta-analyses.

[5] Wang N, Lan D, Chen W, Matsuura F, Tall AR (2004) ATP-binding cassette transporters G1 and G4 mediate cellular cholesterol efflux to high-density lipoproteins. Proc Natl Acad Sci U S A 101:9774-9779

[6] Sturek JM, Castle JD, Trace AP, et al. (2010) An intracellular role for ABCG1-mediated cholesterol transport in the regulated secretory pathway of mouse pancreatic beta cells. J Clin Invest 120:2575-2589

[7] Wu L, Zhu Y (2015) The function and mechanisms of action of LOXL2 in cancer (Review). Int J Mol Med 36:1200-1204

[8] Coral K, Madhavan J, Pukhraj R, Angayarkanni N (2013) High glucose induced differential expression of lysyl oxidase and its isoform in ARPE-19 cells. Curr Eye Res 38:194-203

[9] Stangenberg S, Saad S, Zaky A, Gill A, Pollock CA, Wong MG (2016) LOXL2 as a potential treatment target in diabetic nephropathy. - Poster. (Abstract)

[10] Minn AH, Hafele C, Shalev A (2005) Thioredoxin-interacting protein is stimulated by glucose through a carbohydrate response element and induces beta-cell apoptosis. Endocrinology 146:2397-2405

[11] Shimomura I, Bashmakov Y, Horton JD (1999) Increased levels of nuclear SREBP-1c associated with fatty livers in two mouse models of diabetes mellitus. J Biol Chem 274:30028-30032

[12] Console L, Scalise M, Tarmakova Z, Coe IR, Indiveri C (2015) N-linked glycosylation of human SLC1A5 (ASCT2) transporter is critical for trafficking to membrane. Biochim Biophys Acta 1853:1636-1645

[13] Wahl S, Drong A, Lehne B, et al. (2017) Epigenome-wide association study of body mass index, and the adverse outcomes of adiposity. Nature 541:81-86

[14] Pfeiffer L, Wahl S, Pilling LC, et al. (2015) DNA methylation of lipid-related genes affects blood lipid levels. Circ Cardiovasc Genet 8:334-342

[15] Dekkers KF, van Iterson M, Slieker RC, et al. (2016) Blood lipids influence DNA methylation in circulating cells. Genome Biol 17:138-016-1000-6
